# Treatment in Patients with Psoriatic Disease and Rheumatoid Arthritis: Seven Case Reports

**DOI:** 10.3390/clinpract13010016

**Published:** 2023-01-28

**Authors:** Tomoko Akeda, Keiichi Yamanaka

**Affiliations:** Department of Dermatology, Graduate School of Medicine, Mie University, Tsu 514-8507, Japan

**Keywords:** psoriatic arthritis, rheumatoid arthritis, biologics, methotrexate, TNF-α inhibitor, Il-17

## Abstract

The incidence of psoriasis, an intractable long-lasting inflammatory skin disease, is increasing and has many complications and comorbidities. Approximately 14% of patients have psoriatic arthritis (PsA). Rheumatoid arthritis (RA) is not a rare disease worldwide, and some patients may have both PsA and RA. In the present study, we encountered seven patients with concurrent diagnoses of RA and psoriatic disease and reported the details of clinical data, treatment efficacy, and X-ray findings. The diagnosis may require not only classification criteria but also a comprehensive judgment in collaboration with rheumatology over time. In addition to methotrexate as an anchor drug, anti-tumor necrosis factor-α agents are the first choice of biological agents for treatment, and interleukin (IL)-17 inhibitors may be effective, as IL-17 is also involved in the pathogenesis of RA. When treating patients with both PsA and RA, it may be essential to consider the treatment strategy, depending on which disease is more active.

## 1. Introduction

In Japan, it is estimated that approximately 400,000–500,000 people have psoriasis [1,2], and the average prevalence of psoriatic arthritis (PsA) is reported to be 14.3% [3]. On the other hand, the number of patients with rheumatoid arthritis (RA) in Japan is estimated to be approximately 800,000 [4]. Because RA patients are not uncommon, we may see patients with concomitant RA and PsA in our clinical practice. According to some epidemiological studies, approximately 30% of patients with PsA have RA [5,6,7], and the relative risk of RA in patients is 3.02 times higher than that in healthy individuals [8]. Although the clinical picture of PsA and RA is sometimes very similar, the basic pathogenesis of PsA is enthesitis, which leads to secondary synovitis, whereas that of RA is primary synovitis [9]. The cytokines involved in each disease overlap in some areas but differ in others, and the response to therapeutic agents is also different [10]. Here, we describe seven cases of concurrent diagnoses of RA and psoriatic disease, including clinical data, treatment efficacy, and X-ray findings.

## 2. Cases

### 2.1. Case 1

#### Patient: Man in his 70s

Current medical history: In his 40s, he developed psoriasis and started topical and ultraviolet treatment at his local doctor’s office. In his 50s, he developed arthritis and was diagnosed with RA by an orthopedic surgeon. He was treated with nonsteroidal anti-inflammatory drugs, methotrexate (MTX 4 mg/week), and prednisolone (PSL 7.5 mg/day) for about ten years. In his 70s, he was referred to our department for worsening psoriasis and arthritis.

*Laboratory data:* Antinuclear antibody (ANA) level, 640× (homogenous type); anti-citrullinated protein antibody (ACPA) level, >150 U/mL (normal range, <4.5); rheumatoid factor (RF), 1153 IU/mL (<15); C-reactive protein (CRP) level, 5.2 mg/dL (<0.14); and erythrocyte sedimentation rate (ESR) (1 h), 38 mm/h.

**Figure 1:**
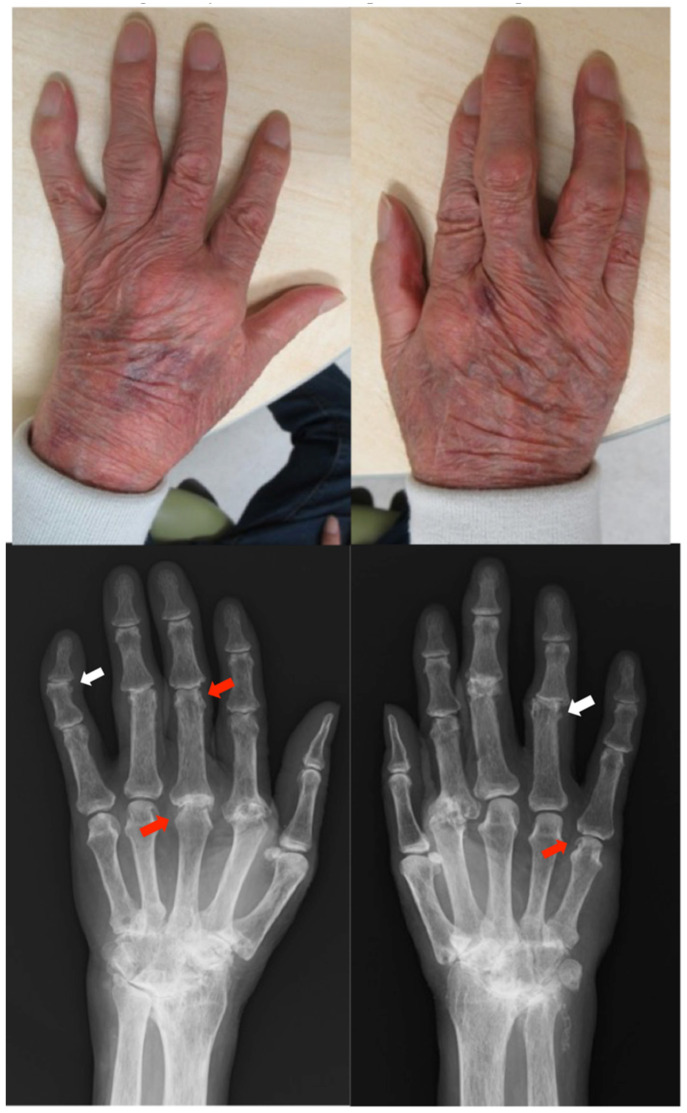
Clinical photographs and radiographs of the hands in case 1. The finger joints are swollen and deformed. Radiographs show symmetrical subluxation of the second MCP joints and bone erosions at the PIP and MCP joints (red arrow). Severe osteolysis and destruction occur on multiple joint surfaces. Severe joint space narrowing and tonic change are depicted in both carpal bones. The left DIP and right PIP joints show bone proliferation suspicious for PsA (white arrow). MCP, metacarpophalangeal; PIP, peripheral interphalangeal; DIP, distal interphalangeal.

*Clinical findings:* The patient had red plaques covered by white scales on the head, trunk, and extremities (PASI score: 17.8) and pain and tenderness in his wrist joints. Radiographs showed symmetrical subluxation of the second metacarpophalangeal (MCP) joint and numerous bone erosions in the bare area (where the synovium is in direct contact with the bone) at the peripheral interphalangeal (PIP) and MCP joints. Severe narrowing of the joint space was observed in the carpal bones. The left distal interphalangeal (DIP) and right PIP joints showed bone proliferation, suggesting PsA (Figure 1). The patient was diagnosed with RA and PsA.

*A course of treatment:* As he had already been treated with MTX (4 mg/week) and PSL (7.5 mg/day) for RA, we started treatment with secukinumab. Four months later, the patient achieved a clear PASI, joint pain disappeared, and the inflammatory reaction was negative.

### 2.2. Case 2

#### Patient: Man in his 60s

*Current medical history:* The patient developed psoriasis at 58 years of age. He had a psoriatic rash on his nails and skin and was treated with topical treatment. At 59 years of age, he developed arthritis and stiffness in his shoulders, neck, wrists, and fingers and was diagnosed with RA by his local physician.

*Past history:* He had a history of iron deficiency anemia (associated with chronic inflammation) and diabetes mellitus.

*Laboratory data:* ANA level, <40×; RF, 279 IU/mL (<15); CRP level, 7.9 mg/dL (<0.14); and ESR (1 h), 85 mm/h.

**Figure 2:**
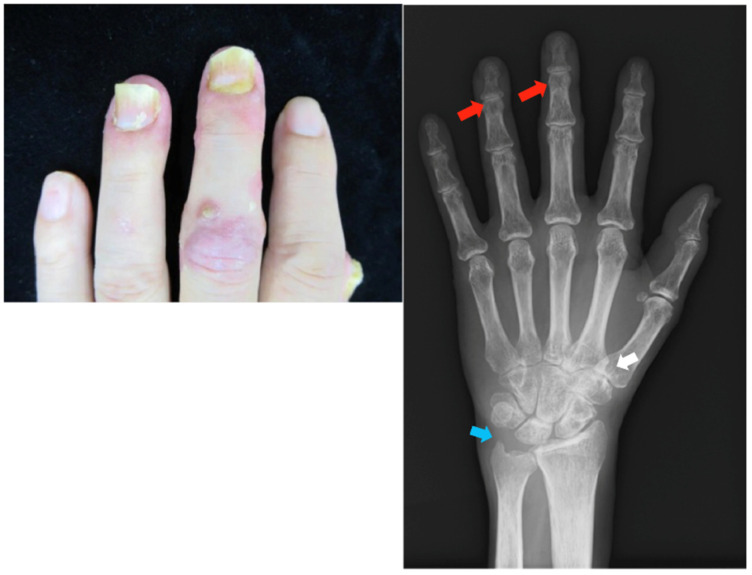
Clinical photograph and radiograph of the hands in case 2. There is nail psoriasis in the left third and fourth fingers and tenderness in the same DIP joints. Joint space narrowing is depicted in the same DIP joints (red arrow). Additionally, joint space narrowing is shown in the left carpal joint (white arrow), and the left styloid process is smaller than the right one (blue arrow). DIP, distal interphalangeal.

*Clinical findings*: There was erythema with white scales on the scalp, dorsum of the hands, and lower extremities (PASI score: 5.2). Onycholysis was observed in the left third and fourth fingers, and tenderness was observed in the same DIP joints. Crumbling was observed in the bilateral thumbs. Radiographs showed joint space narrowing, and the left styloid process was smaller than the right (Figure 2).

*A course of treatment*: Four months after starting treatment with secukinumab, he achieved a clear PASI, the joint symptoms were mild, and the inflammatory response decreased.

### 2.3. Case 3

#### Patient: Woman in her 60s

*Past history*: She had a history of limited cutaneous systemic sclerosis.

*Family history:* Her grandmother had RA.

*Current medical history:* She developed psoriasis at 50 and was treated with topical ointment and oral cyclosporine A (CyA). Her skin rash had worsened at 61 years of age, and she was referred to our hospital (PASI: 26.1). There were red plaques with white scales delineated from surrounding normal skin on the scalp, trunk, and extremities. She participated in a clinical trial of guselkumab for three years and achieved a clear PASI. Following the clinical trial, the medication was switched to secukinumab.

*Laboratory data:* ANA level, 2560× (centromere type); anti-centromere antibody level, >240; ACPA level, 435 U/mL (<4.5); RF, 111 IU/mL (<15); CRP level, 3.26 mg/dL (<0.14), and ESR (1 h), 82 mm.

**Figure 3:**
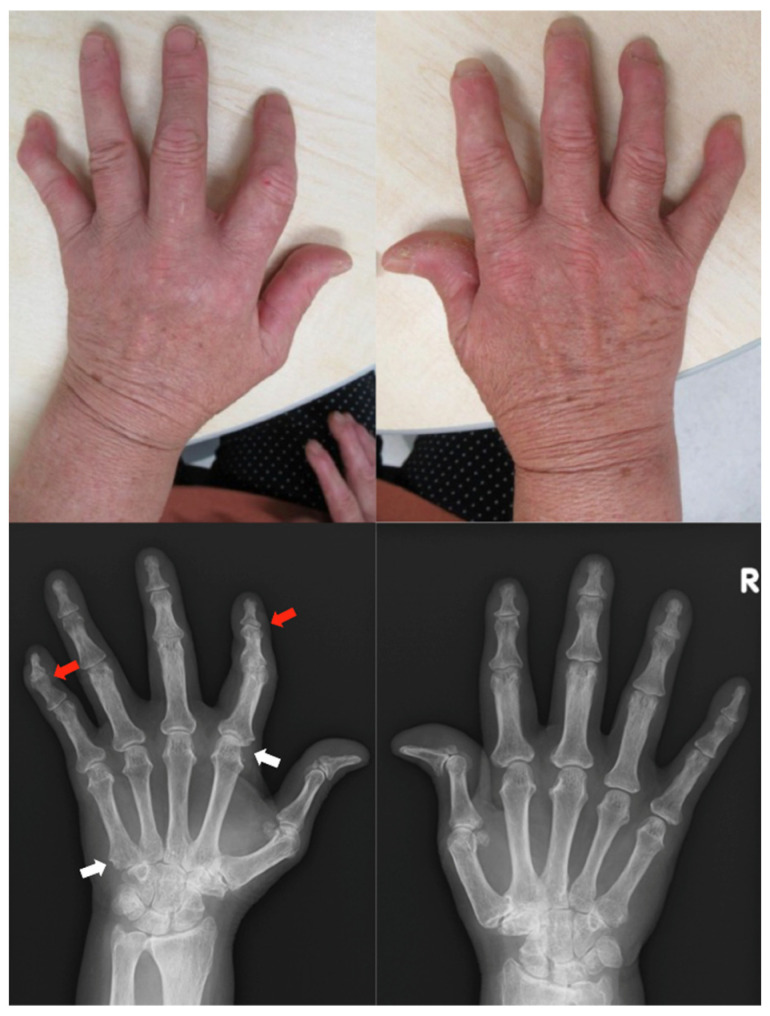
Clinical photographs and radiographs of the hands in case 3. Swan-neck deformities of the left fifth finger and both hitchhiker’s thumbs are shown. Radiographs depict joint space narrowing in the left second and fifth DIP joints (red arrow). Radiographs show subluxation of the left second MCP joints and bone erosions at the left fourth PIP and wrist joint (white arrow). MCP, metacarpophalangeal; PIP, peripheral interphalangeal; DIP, distal interphalangeal.

*Clinical findings:* During nine months of treatment with secukinumab, there was no psoriatic rash, but stiffness of the hands and joint pain appeared in the shoulders and knees. Her hands showed swan-neck deformities of the left fifth finger and both hitchhiker’s thumbs, which are characteristic of patients with RA (Figure 3).

*A course of treatment:* She was treated with MTX (12 mg/week) and PSL (10 mg/day), and the biologics were changed to adalimumab (80 mg/2 weeks). After one year, the CRP level remained >2 mg/dL, and the pain visual analog scale score was 7/10. She was then switched to certolizumab pegol (400 mg/2 weeks), which normalized the inflammatory response and relieved her joint pain after three months. Certolizumab pegol was administered according to the dosage for the treatment of psoriasis.

### 2.4. Case 4

#### Patient: Man in his 50s

*Medical history*: He had an allergic symptom to MTX.

*Current medical history:* Two years ago, he was diagnosed with RA by a local orthopedic surgeon and treated with PSL, MTX, and the anti-IL-6R antibody tocilizumab; the treatment was effective. However, he stopped visiting the hospital because of the coronavirus disease pandemic. Subsequently, arthralgia worsened, and a psoriatic skin rash appeared for the first time.

*Laboratory data:* ANA level, 640× (speckled type); ACPA level, 82.2 U/mL (<4.5); RF, 68 IU/mL (<15); CRP level, 0.36 mg/dL (<0.14); and ESR (1 h), 20 mm.

**Figure 4:**
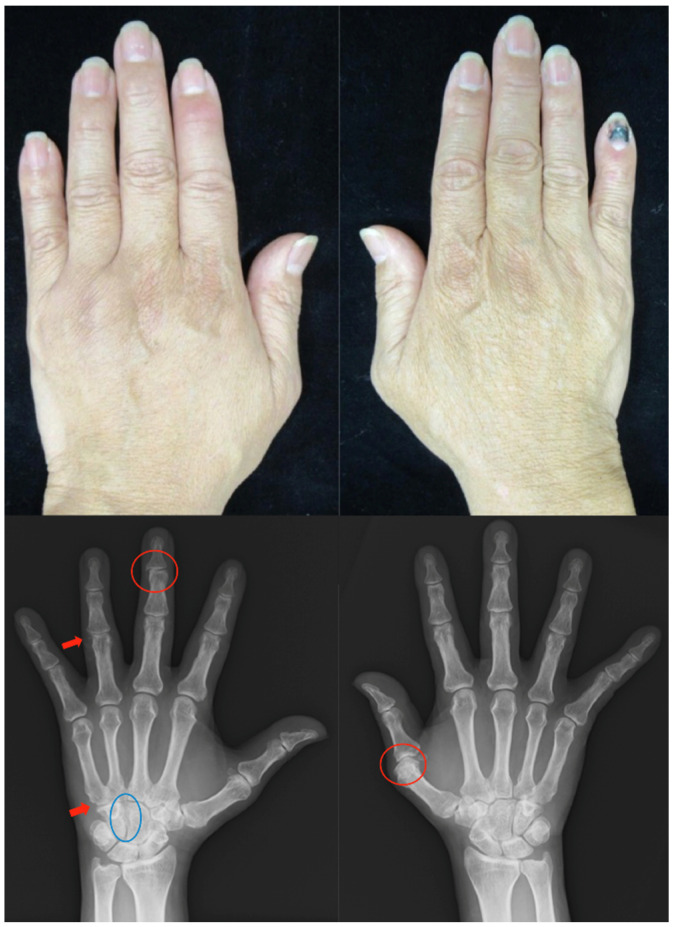
Clinical photographs and radiographs of the hands in case 4. Swelling, pain, and redness are noted in the left second and right fifth joints. There is bone proliferation at the left third DIP and right first MCP joints due to PsA (red circle), marginal erosion in the left fifth proximal metacarpal and fourth PIP joints (red arrow), and joint space narrowing in the left carpal bone (blue circle). MCP, metacarpophalangeal; PIP, peripheral interphalangeal; DIP, distal interphalangeal.

*Clinical findings:* The patient had swelling and pain at the DIP joints of his hands and erythema with fine white scales on the scalp and low limbs (PASI score: 3.6). Pitting and onycholysis were observed on his hand nails. Bone proliferation was observed in the left third and fourth DIP joints due to PsA, marginal erosion in the left fifth proximal metacarpal and right thumb MCP, and narrowing of the joint space in the left carpal bone (Figure 4).

*A course of treatment:* Despite the switch to certolizumab pegol for three months, both inflammatory findings and joint symptoms worsened. Blood tests showed a CRP level of up to 4.05 mg/dL and ESR (1 h) of up to 80 mm. The location of arthralgia expanded to the hand MCP and PIP joints and elbows. The treatment was switched to tocilizumab, which was initially effective, and joint symptoms and inflammatory findings improved.

### 2.5. Case 5

#### Patient: Woman in her 50s

*Past history*: She had a history of hypertension, diabetes mellitus, obesity (body mass index: 38 kg/m^2^), retinal artery occlusion, and interstitial pneumonia (developed during treatment with infliximab and MTX).

*Current medical history:* At 39 years of age, she developed red plaques with silver scales on the trunk and extremities, and was treated with topical ointment only. When she visited our hospital at 52 years old, the psoriatic skin rash gradually worsened, with a PASI score of 11.6. She was treated with CyA (125–200 mg/day) for one and a half years, which was discontinued because of renal failure. At 53 years of age, she developed hand joint pain and onycholysis on her nails and was diagnosed with PsA. After eight months of treatment with infliximab and MTX (6 mg/week), she developed interstitial pneumonia and was switched to ustekinumab. However, ustekinumab for two months did not affect her joint symptoms, so adalimumab was administered for two years, which was effective for both the rash and joint symptoms. At 56 years of age, adalimumab was ineffective.

*Laboratory data:* ANA level, 80× (homogenous type); ACPA level, 55.6 U/mL (<4.5); RF, 34 IU/mL (<15); CRP level, 7.10 mg/dL (<0.14); and ESR (1 h), 95 mm.

**Figure 5:**
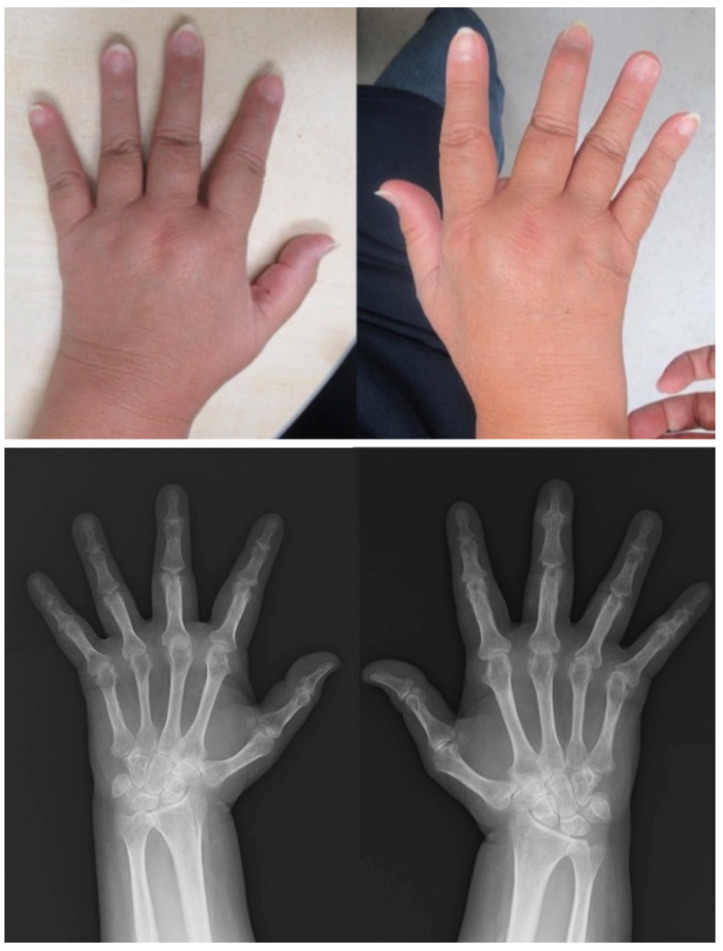
Clinical photographs and radiographs of the hands in case 5. Right-hand fingers show mild ulnar deviation. On the radiographs, the MCP joints are subluxated symmetrically. Radiographs show bone erosion, narrowing joint space, and destruction in both hands’ MCP, PIP, and DIP joints. MCP, metacarpophalangeal; PIP, peripheral interphalangeal; DIP, distal interphalangeal.

*Clinical findings*: During treatment with adalimumab, there was no psoriatic rash, but she had pain in her hand joints, wrists, and knee. Both ACPA and RF values were negative as long as adalimumab was effective; however, when adalimumab became ineffective, both ACPA and RF values were positive with high titers. Radiographs showed bone erosion, a radiolucent shadow, joint destruction in the hand’s MCP, PIP, and DIP joints, bone erosion in the wrist joint, and subluxation of the MCP joint in the ulnar direction (Figure 5).

*A course of treatment:* After two months of treatment with ixekizumab in combination with 1000 mg of salazosulfapyridine and 50 mg of iguratimod, the inflammatory response normalized, and joint pain was relieved. In addition, subluxation in the ulnar direction improved.

### 2.6. Case 6

#### Patient: Man in his 40s

*Past history:* He had a history of hereditary contralateral dyschromatopsia.

*Current medical history:* The patient developed psoriasis at 31 and was treated with MTX (weekly 7.5 mg) and CyA at a nearby hospital. Subsequently, annular erythema with white scales spread to the entire body, and the PASI score worsened to 45, so he was introduced to infliximab. He was well-controlled with infliximab for five years but switched to adalimumab due to secondary failure at 42. After three months of ineffective treatment with adalimumab, he was referred to our hospital. However, we treated with brodalumab with MTX (7.5 mg/week), salazosulfapyridine (1000 mg/day), and PSL (10 mg/day) for four months; secondary failure soon occurred.

*Laboratory data:* ANA level, 80× (homogenous type); RF, 47 IU/mL (<15); ACPA level, 5.8 U/mL (<4.5); CRP, 0.23 mg/dL (<0.14); and ESR (1 h), 10 mm.

**Figure 6:**
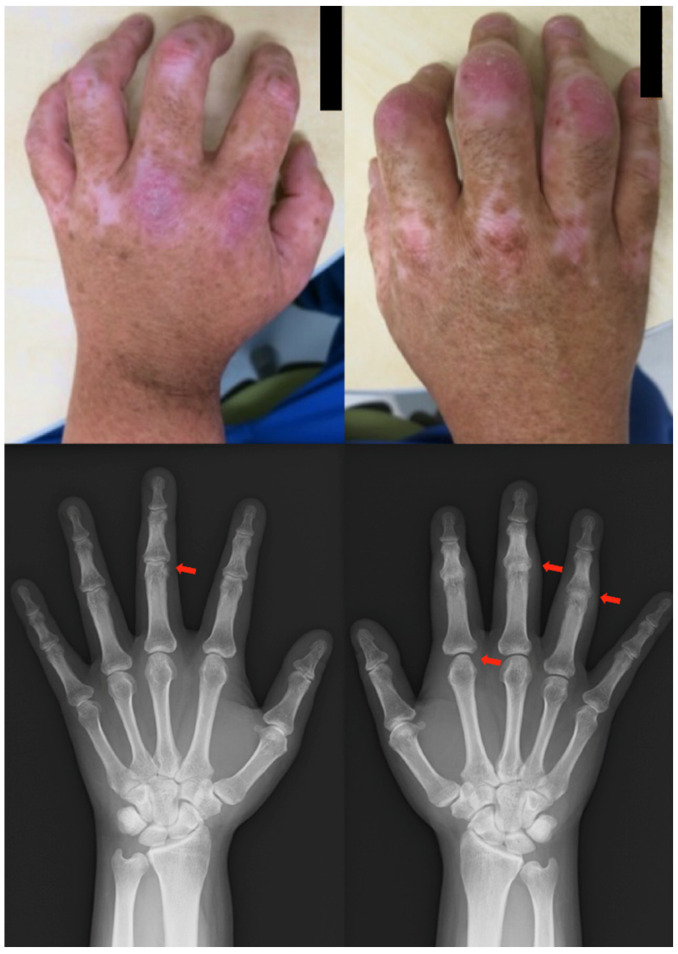
Clinical photographs and radiographs of the hands in case 6. Radiographs show bone erosion, loss of cleft space, and joint destruction in the PIP joint of the hands (red arrow). PIP, peripheral interphalangeal.

*Clinical findings*: Asymmetric swelling and tenderness were noted in both fingers, particularly in the PIP and MCP joints. In addition to the hands, he experienced pain at the enthesis of the knees and ankles, had difficulty walking, and was confined to a wheelchair. The RF value was initially negative, but it began to show strong positivity from this time onward, exceeding three times the standard value. Radiographs showed bone erosion, loss of cleft space, and destruction of the PIP joint of the hands (Figure 6).

*A course of treatment:* The patient was switched to ixekizumab, and concomitant medication included MTX (14 mg/week), salazosulfapyridine(1000 mg/day), and PSL (10 mg/day). After three months, the inflammatory reaction was negative, and the patient recovered to walking and activities of daily living without any problems. Additionally, subluxation in the ulnar direction occurred during the process, but it was improved.

### 2.7. Case 7

#### Patient: Man in his 50s

*Past history:* He had a history of gout.

*Current medical history:* At 50 years of age, he developed RA and was treated with adalimumab, MTX (6 mg/week), and abatacept or adalimumab by his local rheumatologist. At 55 years of age, red plaques with fine scales appeared on his scalp and extremities, so he was referred to our hospital.

*Laboratory data:* ANA level, <40×; RF, 13 IU/mL (<15); ACPA level, 787.9 U/mL (<4.5); CRP level, 0.05 mg/dL (<0.14); and ESR (1 h), 12 mm.

**Figure 7:**
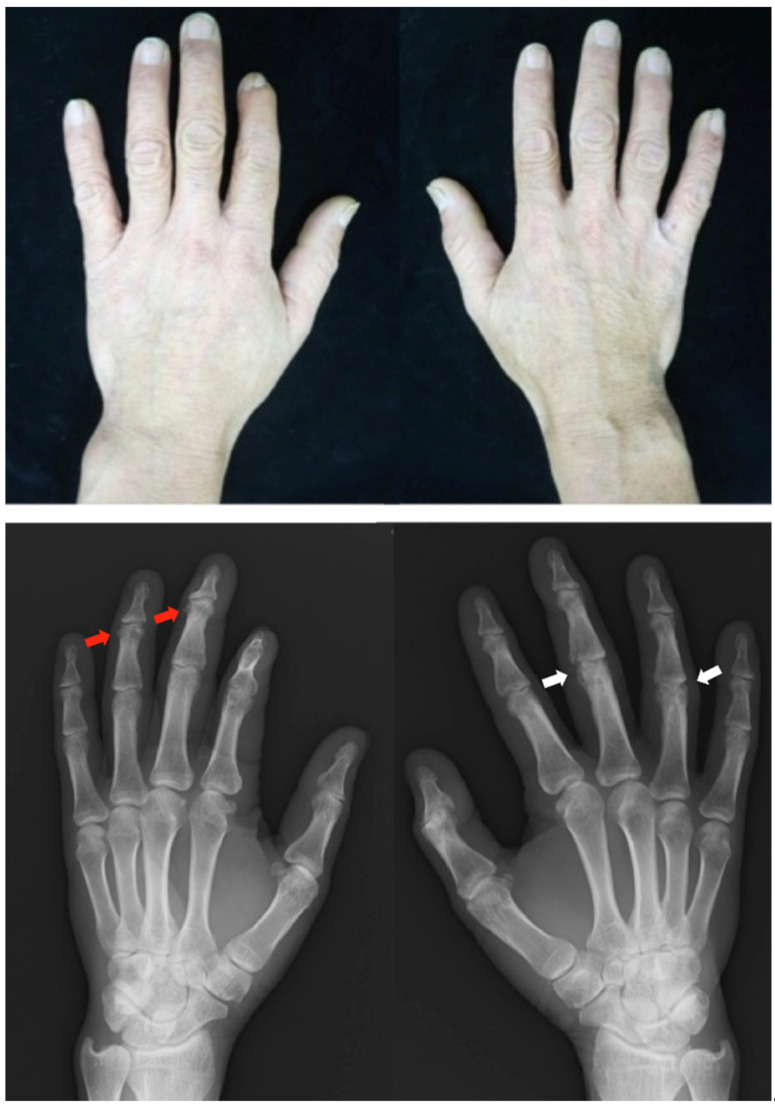
Clinical photographs and radiographs of the hands in case 7. Radiographs show erosions adjacent to periarticular bone proliferation at the collateral ligament and capsular joint attachment site (red arrow). Bone erosions are noted in the right third and fourth PIP joints (white arrow). PIP, peripheral interphalangeal.

*Clinical findings*: The patient had psoriatic plaques on the scalp, lower legs (PASI score: 9.8), and ankle and hand arthralgia. Onycholysis was observed on his hand nails. Radiographs of the hands showed narrowing of the joint space and destruction in the DIP and PIP joints (Figure 7).

*A course of treatment:* After five months of administration of ixekizumab in combination with MTX (6 mg/week), the skin rash and arthralgia disappeared.

### 2.8. Summary of the Cases

Of the seven patients in the current study, five were male, and two were female. The mean age at the onset of joint symptoms was 50.3 years. All patients met the RA classification criteria, and six met the Classification Criteria for Psoriatic Arthritis (CASPAR) classification. The male-to-female ratio in RA is said to be approximately 1:3. In psoriasis, it is reported to be approximately 2:1 [11]. In our cases, the male-to-female ratio was 5:2. Since our cases were reported in arthritis patients who came to the dermatologists for psoriasis treatment, we think that the distribution of the gender ratio was closer to that of psoriasis patients than RA.

MTX was used in six out of the seven patients. The anti-tumor necrosis factor (TNF)-α antibody was used in seven patients and was effective in five; the anti-IL-17 antibody was used in seven patients and was effective in five, and the anti-IL-23 antibody was used in one patient, but there was no response. The anti-IL-6R antibody was successfully used in one case, and CTLA4-Ig was used in one case without success. A summary of these cases is provided in Table 1. The disease activity to determine treatment efficacy was evaluated using the PASI score for psoriasis skin symptoms, SDAI (Simplified Disease Activity Index) for rheumatoid arthritis, and DAPSA (disease activity index for psoriatic arthritis) for PsA joint symptoms.

## 3. Discussion

RA is an autoimmune disease that involves multiple joints bilaterally. It is characterized by tenosynovitis [12]. In contrast, psoriasis is a chronic and intractable inflammatory skin disease; it is a well-known fact that cutaneous inflammation is not only a localized problem of the skin but also causes inflammation of the organs throughout the body, resulting in comorbidities and a shortened life expectancy [13]. Cerebral and cardio-arteriosclerosis, adipose tissue remodeling, osteoporosis, male infertility, and gastrointestinal amyloidosis are found to be closely related to a long-lasting inflammatory skin disease mice model [14,15,16,17,18,19,20,21]. In recent years, psoriasis has attracted particular interest because of its association with some comorbidities, such as cardiovascular disease and its risk factors: obesity, arterial hypertension, dyslipidemia, and type-2 diabetes mellitus, and its diagnosis and management have been discussed. The relationship between psoriasis and enthesitis is one of them [22]. PsA is a seronegative arthropathy in which inflammation begins as enthesitis and spreads to the synovium, causing secondary synovitis [9].

Like PsA, RA is also associated with an increased risk of comorbidities related to systemic inflammation (e.g., cardiovascular disease) [23,24]. Han et al. found a similarly increased prevalence of ischemic heart disease, atherosclerosis, peripheral vascular disease, congestive heart failure, cerebrovascular disease, hyperlipidemia, and hypertension in RA and PsA patients compared to healthy controls [25]. Interstitial lung disease (ILD) is the most common and severe comorbidity of RA, and lymphoproliferative diseases have also been reported [26]. Complications of uveitis, inflammatory bowel disease, and non-alcoholic fatty liver disease (NAFLD) have also been reported in PsA [27]. When both diseases are combined, attention should be paid to the common cardiovascular disease and the specific comorbidities of each.

PsA and RA are common chronic inflammatory diseases characterized by joint pain and swelling with significant systemic manifestations. Delayed diagnosis and treatment can lead to joint destruction with loss of function, significantly reducing the patient’s quality of life. Neither PsA nor RA have diagnostic criteria that can be applied to individual patients. The diagnosis and differentiation are based on the CASPAR and the American College of Rheumatology/European League Against Rheumatism classification criteria. The serological tests RF/ACPA are also included in the classification criteria; however, positive or negative results do not exclude the diagnosis of PsA or RA. Approximately 80% of patients with RA have positive RF or ACPA results [28,29]. However, PsA is seronegative arthritis, and RF/ACPA is basically negative; it is estimated that approximately 10% of PsA cases are RF/ACPA-positive, and titers are usually low [28,29,30,31]. A study comparing patients with RA or PsA with controls reported that the mean RF and ACPA titers in patients with RA were much higher than those in patients with PsA (RF titer: 56 versus [vs.] 11 U/mL; ACPA titer: 14 vs. 2 U/mL) [29]. The presence of RF antibodies or ACPA in serum also suggests that if the ACPA titer is >11.6 U/mL, the patient is more likely to have RA than PsA [29]. In our case, both RF and ACPA titers were positive, or one of them was positive with high titers, supporting the presence of RA.

In RA, early treatment with MTX plus glucocorticoids and subsequently with other disease-modifying anti-rheumatic drugs (DMARDs), such as inhibitors of TNF-α, IL-6, or Janus kinases, improves outcomes and prevents RA-related disability [12]. In contrast, inhibition of IL-17, IL-23, and TNF-α, the major effector cytokines of ligament inflammation in PsA, is effective in supporting the resolution of ligament inflammation in PsA [9]. MTX was used in six cases in this study. MTX has become the “anchor drug” for RA. MTX has greater efficacy and effectiveness than any other non-biological DMARD and has more excellent tolerability and safety than other DMARDs [32]. Therefore, when PsA is associated with RA, as in our cases, MTX administration may be considered first. TNF-α is deeply involved in the pathogenesis of both RA and PsA, and anti-TNF-α antibodies, such as infliximab, adalimumab, and certolizumab pegol, are effective and recommended for both diseases [33,34]. Therefore, anti-TNF-α antibody agents can be the first choice when considering the administration of biological agents in patients with RA and PsA, as in the present cases. Additionally, IL-17 is deeply involved in the pathogenesis of PsA, and anti-IL-17 agents, such as secukinumab, ixekizumab, and brodalumab, are highly effective in treating both psoriatic rash and joint symptoms of PsA. However, they are not recommended for the treatment of RA because they have shown only modest efficacy compared with their effect on PsA in clinical trials [34,35]. However, these results do not imply that IL-17 is not involved in the pathogenesis of RA. The presence of IL-17A and IL-17A-producing CD4^+^ T cells in rheumatoid joints confirms the biological function of IL-17A in promoting inflammation, angiogenesis, and osteoclast genesis [35]. We believe that the fact that IL-17 plays an important role in RA is one of the reasons for the success of anti-IL-17 antibodies in many of the cases of RA that we experienced here. In addition, two patients developed and recovered from subluxation in the ulnar direction, a symptom specific to RA. No particular medication was changed at the onset, but spontaneous recovery was observed approximately six months after onset. The initial subluxation in the ulnar direction was reversible. Although the number of reported cases of RA and PsA was small in this study, we think that the outcome of whichever drug was responsive depends on which was predominant, at the time, in the activity of PsA or RA.

## 4. Conclusions

Here, we reported the treatment of patients with RA and PsA. Based on these cases, MTX is an effective treatment option. Depending on the subject, TNF-α, IL-17, and IL-6 inhibitors may effectively treat joint symptoms. This difference may depend on whether PsA or RA is predominant at the time of treatment.

## Figures and Tables

**Table 1:** Summary of clinical findings, laboratory data, and therapies of seven patients with concomitant rheumatoid arthritis and psoriatic arthritis.

Patient		Psoriatic Skin Lesion		Joint Involvement			Laboratory Data		Therapy	
	Age/Sex	Duration (Years)	Nail	Duration (Years)	Axial	CASPAR ≥ 3	RF	ACPA	Systemic	Biologic Agent
1	77/M	30	-	33	-	+	1100	150	NSAIDs, MTX, & PSL	SEC
2	60/M	2	+	1	+	+	279	NA	NSAIDs	SEC
3	67/F	18	-	2	-	N	100	435	MTX, PSL, & CyA	ADA & CZP GUS, SEC,
4	58/M	1	+		-	+	68	88.2	NSAIDs, MTX, & PSL	CZP, & TCZ
5	57/F	20	+	8	-	+	30	55.6	NSAIDs, MTX, SASP, & IGU	IFX, ADA, & IXE
6	47/M	16	-	10	-	+	negative to 7	5.8	NSAIDs, MTX, PSL, SASP, & CyA	IFX, BRO, & IXE
7	56/M	3	+	8	-	+	negative	787.9	NSAIDs & MTX	ADA, ABA, & IXE

Underlined drugs were clinically successful in the treatment of joint symptoms. SASP, salazosulfapyridine; SEC, secukinumab; ADA, adalimumab; CZP, certolizumab pegol; GUS, guselkumab; TCZ, tocilizumab; UST, ustekinumab; IXE, ixekizumab; IFX, infliximab; BRO, brodalumab; ABA, abatacept; F, female; M, male; NSAIDs, non-steroidal anti-inflammatory drugs; MTX, methotrexate; CyA, cyclosporine A; PSL, prednisolone; IGU, iguratimod; CASPAR, Classification Criteria for Psoriatic Arthritis.

## Data Availability

The patient’s data is not publicly available on legal or ethical grounds. Further inquiries can be directed to the corresponding author.
